# Phenylboronic Acid and Amino Bifunctional Modified Adsorbent for Quickly Separating Phenolic Acids from Crude Extract of *Clerodendranthus spicatus* and Evaluation of Their Antioxidant and Hypoglycemic Activities

**DOI:** 10.3390/molecules28227539

**Published:** 2023-11-11

**Authors:** Mengqi Cheng, Qianyi Song, Xiaoyu Zhang, Pingyi Zheng, Ran Zhao, Youxin Li, Haixia Chen

**Affiliations:** Tianjin Key Laboratory for Modern Drug Delivery and High-Efficiency, Collaborative Innovation Center of Chemical Science and Engineering, School of Pharmaceutical Science and Technology, Tianjin University, Tianjin 300072, China; chengmengqi@tju.edu.cn (M.C.); songqy98@tju.edu.cn (Q.S.); 15153501767@163.com (X.Z.); m18804096535_1@163.com (P.Z.); zhaoran_97@163.com (R.Z.)

**Keywords:** phenolic acids, phenylboronic acid, amino, adsorbents, *Clerodendranthus spicatus*, antioxidant activity, hypoglycemic effect

## Abstract

A novel phenylboronic acid and amino bifunctional modified silica gel (SiO_2_-NH_2_-FPBA) was prepared, which was 30–80 μm, had a pore size of 8.69 nm, a specific surface area of 206.89 m^2^/g, was stable at low temperature, and contained 0.4793 mmol/g of the phenylboronic acid group and 1.6377 mmol/g of the amino group. It was used to develop a rapid separation method for phenolic acids. The results showed that it could adsorb 93.64 mg/g caffeic acid, 89.35 mg/g protocatechuic acid and 79.66 mg/g gallic acid. The adsorption process was consistent with the pseudo-second-order model (R^2^ > 0.99), and fitted the Langmuir isotherm model well (R^2^ > 0.99). CH_3_COOH could effectively desorb phenolic acids (>90%) and did not destroy their structures. When SiO_2_-NH_2_-FPBA was added to crude extract of *Clerodendranthus spicatus,* 93.24% of the phenolic acids could be captured, and twenty-two kinds of phenolic acids were identified by Q Exactive HF LC-MS. Furthermore, the isolated phenolic acids from *Clerodendranthus spicatus* possessed great DPPH, ABTS, and hydroxyl radicals scavenging activities and ferric reducing power. They also demonstrated effective inhibition of α-amylase and α-glucosidase activities (IC_50_ = 110.63 ± 3.67 μg/mL and 64.76 ± 0.30 μg/mL, respectively). The findings indicate that SiO_2_-NH_2_-FPBA has significant potential in practical applications of separating active constituents from natural resources.

## 1. Introduction

Phenolic acids are widely distributed in medicinal plants and are secondary metabolites produced by the plants themselves, accounting for one-third of the polyphenolic compounds [1]. Phenolic acid compounds are mainly composed of two carbon skeletons, respectively—C6-C1 benzoic acid type (e.g., gallic acid) and C6-C3 cinnamic acid type (e.g., caffeic acid). The different positions and number of hydroxyl groups on the aromatic ring and the various substituents on the carbon positions of the aromatic ring allow phenolic acid to form different kinds and structures of compounds. Phenolic acid substances have strong biological activities, including antitumor, antioxidant, anti-inflammatory, antibacterial and antiviral activity [2,3,4], and have been extensively studied in various fields, such as agriculture, food science, medicine, and environmental science. Taking into account further research into the pharmacological activities, sources (other traditional Chinese medicines) and applications of phenolic acids, developing an effective method for the fast separation of phenolic acids is becoming an important issue.

In the past few decades, many methods have been developed for the separation of phenolic acids, such as precipitation [5], membrane separation [6], extraction [7], chromatography [8,9], solid phase adsorption [10] and resin [11]. The most commonly used extraction method is solvent extraction to obtain the crude extracts, followed by separation of the extract using chromatographic techniques through a resin adsorption column [12]. For example, Sun et al. [13] chose Sephadex LH-20 column chromatography and reverse phase C18 gel column chromatography and Zhou et al. [14] used HW-40C and LH-20 column chromatography for the separation and collection of phenolic acids. The resin adsorption method has the advantages of being a simple process, less energy consumption and green efficiency. However, this technique has presented a number of issues such as excessive loss of solvents, inadequate separation efficiency, residual solvents, and operational hazards. Furthermore, it was limited in small-scale sample preparation and was unsuitable for large-scale industrial applications [12].

Remarkably, the adsorption method has gained a lot of attention due to its high efficiency, low cost, ease of operation, and reproducibility [15]. Various types of adsorbents have been used for phenolic acids’ adsorption. For example, Cagnon [16] investigated the adsorption of gallic acid on an activated carbon oxidized by ozone. Li [17] and Fan [18] utilized molecularly imprinted materials for the isolation and purification of caffeic acid from mushrooms and fruits. Song [19] and Chai [20] prepared magnetic chitosan-modified diatomite to adsorb gallic acid and caffeic acid from a sugar solution. Nie [21] and Du [12] synthesized ionic liquid silica gel for the adsorption and separation of water-soluble phenolic acids. Moritz et al. [22] synthesized mesoporous SBA-15 and MCF silica functionalized with aminopropyltriethoxysilane (APTES) and 3-[2-(aminoethylamino) propyl] trimethoxy silane (AEAPTMS) and explored their adsorption effect on caffeic acid. Simanaviciute et al. [10] prepared cationic cross-linked starch for adsorbing caffeic, chlorogenic and rosmarinic acids. However, most of these adsorption methods rely on electrostatic attraction to exert the adsorption effect. Therefore, considering the chemical properties of phenolic acids, which have a specific structure of cis-diols that can react with boronic acids in a specific way, boronic-acid-modified adsorbents are considered ideal for preparation. For instance, Qian [23] used 4-(acryloyloxy)phenylboronic acid as a monomer to prepare a surface molecularly imprinted magnetic nanoparticle for recovering salvianolic acid. Su [24] prepared polyethyleneimine intercalated montmorillonite with phenylboronic acid groups for capturing salvianolic acid A, salvianolic acid B and rosmarinic acid. It is worth noting that the formation of borate is reversible and is affected by the pH of the solution based on the reaction mechanism of boric acid and cis-diol [23]. It is stable under weakly basic conditions (pH ≥ 8.5). Nevertheless, phenolic acids can make the solution acidic after ionization, which hinders the combination of boric acid and phenolic acids. Therefore, the addition of basic groups is particularly important, which can neutralize the acidity of phenolic acids and promote the formation of borate.

*Clerodendranthus spicatus* (*C. spicatus*), commonly known as “Shen Cha” in Chinese, is a perennial herb that grows widely in tropical and subtropical regions [14,25,26]. It has been used to treat diabetes and kidney diseases, such as urinary lithiasis, chronic nephritis, urinary tract infection, nephrotic syndrome, and chronic renal failure with a long history. More than 150 kinds of compounds have been isolated from *C. spicatus*, and the main chemical substances are phenolic acids, diterpenoids, triterpenoids, and their derivatives [27]. Over 60 kinds of phenolic acids and their derivatives have been separated by traditional methods, including protocatechuic acid [28], caffeic acid [28], and their derivatives, among others [13]. However, there is no information about the rapid separation of phenolic acids from crude extract of *C. spicatus* by special adsorbents.

In this study, we synthesized a novel phenylboronic acid and amino bifunctional modified silica gel (SiO_2_-NH_2_-FPBA) using surface modification methods. It was systematically characterized by fourier transform infrared spectroscopy (FT-IR), scanning electron microscopy (SEM), energy dispersive spectrometry (EDS), transmission electron microscopy (TEM), thermogravimetric analysis (TGA), the Brunauer–Emmett–Teller (BET) method, and inductively coupled plasma-optical emission spectrometry (ICP-OES). Three common phenolic acids—gallic acid (GA), caffeic acid (CA) and protocatechuic acid (PCA)—were selected as the model target, and the effects of different adsorption conditions were investigated and optimized. On the basis of these, the method was applied for the first time for one-step and rapid separation of phenolic acids from *C. spicatus* crude extract. Then, the antioxidant activity and hypoglycemic activity of the isolated phenolic acids from *C. spicatus* were determined by 1,1-diphenyl-2-picrylhydrazyl (DPPH), 2,2′-azinobis-(3-ethylbenthiazoline-6-sulfonate) (ABTS), hydroxyl radicals scavenging assays, ferric-reducing antioxidant activity, and α-amylase and α-glycosidase inhibition effects in vitro. This study provided a new and rapid method for the separation of phenolic acids from traditional Chinese medicine.

## 2. Results and Discussion

### 2.1. Characterization

**Figure 1:**
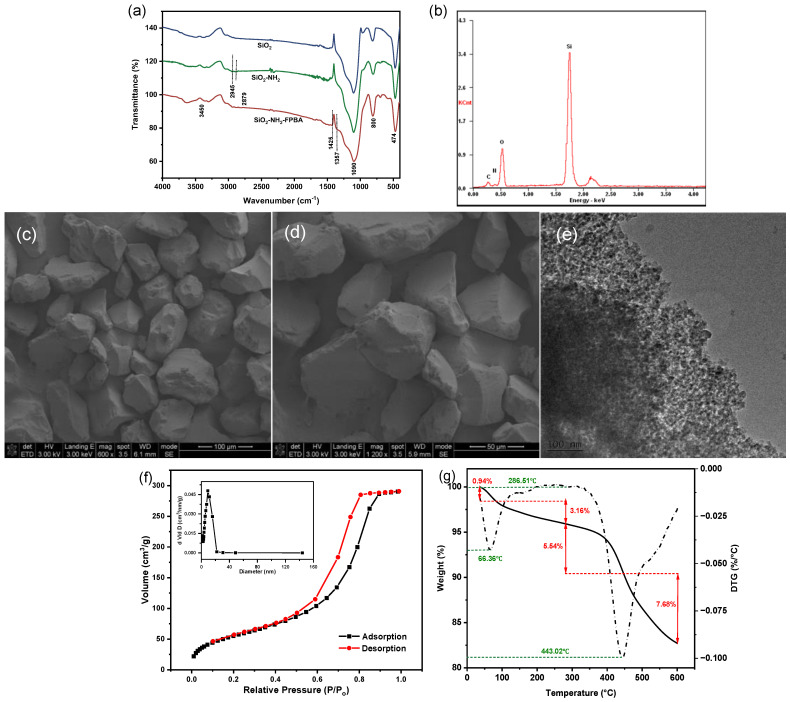
Characterization of phenylboronic acid and amino bifunctional modified silica gel (SiO_2_-NH_2_-FPBA): (**a**) The FT-IR spectra of SiO_2_-NH_2_-FPBA and synthetic intermedia; (**b**) The EDS image; (**c**–**e**) The SEM and TEM images; (**f**) Nitrogen adsorption–desorption isotherm; (**g**) Thermogravimetric curve.

FT-IR was used to characterize the presence of the corresponding functional groups on the SiO_2_-NH_2_-FPBA surface (Figure 1a). The peaks at 3450 cm^−1^, 1090 cm^−1^ and 800 cm^−1^ were the characteristic absorptions formed by the stretching vibration of -OH, asymmetric stretching vibration of Si-O-Si and bending bands of Si-OH, respectively [29]. The peaks at 2945 cm^−1^ and 2879 cm^−1^ were attributed to the asymmetric and symmetric stretching vibration of methylene [30,31], indicating that the amino groups were successfully attached and providing the amino sites for the next reaction [32]. The absorption peak at 1425 cm^−1^ indicated the skeleton vibration of the benzene ring and 1357 cm^−1^ was formed by B-O stretching vibration, which indicated that the phenylboronic acid had been successfully attached.

The surface was elementally analyzed by EDS and is shown in Figure 1b, containing 36.91% Si element, 39.09% O element, 3.94% N element and 20.05% C element. The response of the B element was low using this method and could not be clearly shown. The morphology and structure of the SiO_2_-NH_2_-FPBA were observed by SEM and TEM (Figure 1c,d). It could be seen that the material showed an irregular shape and the particle size was 30–80 μm. When enlarged, it could be found that the surface was rough and uneven with pores and concavities, which effectively provided a large number of adsorption sites and facilitated the adsorption. The light and dark areas in Figure 1e indicate the existence of mesopores in the SiO_2_-NH_2_-FPBA.

The adsorption and desorption isotherms of N_2_ and the pore size distribution curve of SiO_2_-NH_2_-FPBA are shown in Figure 1f. The N_2_ adsorption of SiO_2_-NH_2_-FPBA was a typical IV isotherm [33,34]. Its hysteresis loop appeared between 0.40 and 0.90, which belonged to the H1 type with disordered pores [33]. The pore size of 8.69 nm indicated SiO_2_-NH_2_-FPBA with mesopores [31]. The BET specific surface area of SiO_2_-NH_2_-FPBA calculated from the nitrogen adsorption analysis was 206.89 m^2^/g.

The TGA curve of SiO_2_-NH_2_-FPBA is shown in Figure 1g. A small weight loss of 0.94% for the material was found due to the evaporation of water [35]. At slightly greater than the boiling point of phenylboronic acid (265.9 °C), a weight loss of 3.16% at 286.51 °C was produced, presumably due to phenylboronic acid degradation. A weight loss of 5.54% in the range of 286.51–443.02 °C and 7.68% loss when temperature was up to 600 °C were observed, which may be attributed to the decomposition of the amino-propyl group on the surface and the dehydration condensation of the -OH on the silica gel surface [36]. The SiO_2_-NH_2_-FPBA was thermostable at a low temperature.

ICP-OES data showed that the amount of B in SiO_2_-NH_2_-FPBA was 0.4793 mmol/g, which indicated that the B element was attached to the material surface. The content of the residual amino group in SiO_2_-NH_2_-FPBA reduced from 2.1170 mmol/g in SiO_2_-NH_2_ to 1.6377 mmol/g. The ratio of phenylboronic acid group to amino group was about 3.4:1.

### 2.2. Evaluation of the Adsorption Ability of SiO_2_-NH_2_-FPBA to GA, CA and PCA

#### 2.2.1. Effect of pH on Absorption

**Figure 2:**
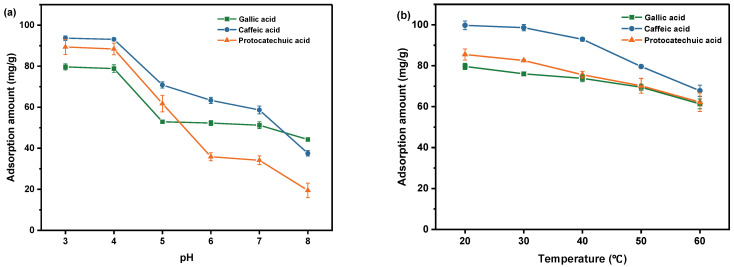
Effects of different factors on the adsorption capacity of three phenolic acids: (**a**) pH; (**b**) temperature.

To explore the effect of pH, the GA, CA and PCA solutions were adjusted to 3, 4, 5, 6, 7, and 8 using HCl or NaOH. SiO_2_-NH_2_-FPBA (20 mg) was added into 8 mL of 0.5 mg/mL solution of GA, CA and PCA at different pH values. Batch adsorption experiments were conducted in a temperature-controlled water bath oscillator at 200 rpm at 20 °C for 4 h. At the end of the adsorption, the solid was separated from the liquid by centrifugation at 4100 rpm for 3 min. The concentration of each solution in the supernatant was measured by UV, and the results are shown in Figure 2a. It was found that the adsorption effect was best at pH 3, with 93.64 mg/g for CA, 89.35 mg/g for PCA and 79.66 mg/g for GA. When the pH was increased to 4, the adsorption amount changed slightly. The adsorption produced a significant decreasing trend when the pH was increased from 4 to 8. As is known, the pKa values of GA, CA and PCA were 4.41, 4.58 and 4.48, respectively, which existed in ionic form and formed sodium phenol at high pH. The instability of the three phenolic acids at higher pH seemed to be the reason for their significantly lower adsorption [19,24]. The pH values of the aqueous solutions of the three phenolic acids at 0.5 mg/mL were determined to be 3.39, 3.43 and 3.44, respectively, which were between the optimum pH of 3 and 4. Therefore, aqueous solutions of phenolic acids were used for adsorption in the next experiments.

#### 2.2.2. Effect of Temperature on Absorption

Adsorption temperature may affect the adsorption performance of phenolic acid substances. To explore the effect of temperature, 20 mg of SiO_2_-NH_2_-FPBA was added into 8 mL of 0.5 mg/mL of aqueous solution of GA, CA, or PCA. The centrifuge tubes were placed in a water bath with different temperatures (20, 30, 40, 50, and 60 °C). From Figure 2b, it can be seen that with the increase in adsorption temperature from 20 °C to 60 °C, their adsorption amount decreased from 99.75 mg/g to 67.78 mg/g, 85.48 mg/g to 62.19 mg/g and 79.66 to 61.36 mg/g, respectively, indicating that the increase in temperature was unfavorable for the adsorption process. Thus, all the following experiments were conducted at 20 °C.

#### 2.2.3. Effect of Time on Absorption

**Figure 3:**
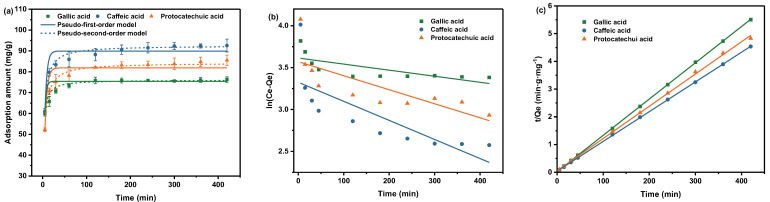
(**a**) Adsorption kinetics of SiO_2_-NH_2_-FPBA for three phenolic acids and the model fitting of the pseudo-first-order and pseudo-second-order equations. Fitted curves of (**b**) pseudo-first-order models and (**c**) pseudo-second-order models of the adsorption.

Adsorption kinetics can provide valuable information on solute uptake rate and additional details on the overall adsorption process [37]. To explore the adsorption kinetics, 20 mg of SiO_2_-NH_2_-FPBA was added into 20 mL of 0.5 mg/mL aqueous solution of phenolic acids at 20 °C. The supernatant was sampled at 5, 15, 30, 60, 120, 180, 240, 300, 360, and 420 min and the adsorption amount was measured. The results shown in Figure 3a indicate that the adsorption started at a fast rate, then the adsorption rate gradually slowed down. With the extension of adsorption time, the adsorption capacity kept increasing. From 0 to 120 min, the adsorption amount gradually increased over time due to the presence of a large number of adsorption sites for adsorption in the initial stage. At the 120–420 min stage, the adsorption gradually tended toward equilibrium and reached saturation with the adsorption amounts of 92.58 mg/g for CA, 85.54 mg/g for PCA and 76.25 mg/g for GA.

To further analyze the adsorption of SiO_2_-NH_2_-FPBA to phenolic acids, the pseudo-first-order and pseudo-second-order kinetic models were exploited to fit the experimental data. The equations of the two models were as follows:

The pseudo-first-order equation was

(1) $$ln\left(Q_{e}-Q_{t}\right)=lnQ_{e}-K_{1}t$$

The pseudo-second-order equation was

(2) $$\frac{t}{Q_{t}}=\frac{t}{Q_{e}}+\frac{1}{{K_{2}\times Q}_{e}^{2}}$$

where K_1_ (min^−1^) and K_2_ (g(mg/min)) are the pseudo-first-order rate constant and pseudo-second-order rate. Q_t_ (mg/g) and Q_e_ (mg/g) represent the adsorption amount for each phenolic acid at t time and equilibrium, respectively.

The fitting curves are shown in Figure 3b,c and the constants are summarized in Table 1. It was found that the pseudo-second-order model had better agreement; all four R^2^ values were above 0.999. The adsorption processes of the three phenolic acids could be well represented by pseudo-second-order kinetics, which demonstrated that there was a rate-limiting step in the adsorption process. This was a chemical adsorption process.

#### 2.2.4. Effect of Concentration on Absorption

The adsorption isotherm can be used to not only assess the adsorption capacity of adsorbents, but also can describe how the adsorbate interacts with the adsorbent [38]. Thus, the effect of concentration on adsorption was investigated. Each of the three solutions was prepared as 0.1, 0.25, 0.5, 1, 2.5, and 5 mg/mL. Then, 20 mg of each adsorbent was added into 8 mL of different solutions at 20 °C for 2 h. To understand the relationship between Q_e_ and C_e_ under equilibrium conditions, the adsorption isotherms were analyzed by obtaining the best fits to the data using the extensively used Langmuir and Freundlich models. The Langmuir model assumes a monolayer adsorption, which means that adsorption molecules adsorbed on the surface of the adsorbent have the same adsorption activation energy. It assumes that the adsorbate and adsorbent are in an ideal state and the model is deployed for homogenous surfaces. The Freundlich model assumes a multilayer adsorption, which means there are many adsorption sites on the adsorbent, and the adsorption sites have different free energy values, which can adsorb multiple molecules. This model is applicable in the study of adsorption on rough and multisite (heterogonous) surfaces. These two models can be expressed using Equations (3) and (4):

(3) $$\frac{C_{e}}{Q_{e}}=\frac{1}{bQ_{0}}+\frac{C_{e}}{Q_{0}}$$

(4) $$\ln Q_{e}=\ln K_{f}+\frac{1}{n}\ln C_{e}$$

where Q_e_ is the adsorption capacity at equilibrium (mg/g), C_e_ is the concentration of the solution at equilibrium (mg/mL), Q_0_ is the saturation adsorption capacity (mg/g), b represents the equilibrium adsorption constant and the adsorption affinity, n is the Freundlich constant, and K_f_ is the binding constant.

**Figure 4:**
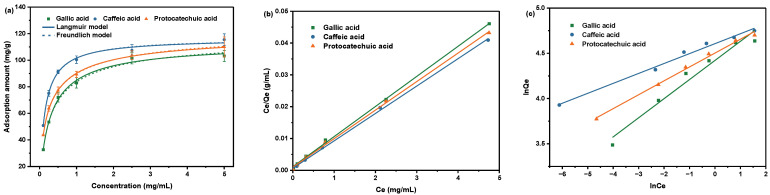
(**a**) Adsorption isotherm of SiO_2_-NH_2_-FPBA for three phenolic acids and the model fitting of the Langmuir and Freundlich models. Fitted curves of (**b**) Langmuir model and (**c**) Freundlich model of the adsorption.

From Figure 4a, it is obvious that the adsorption amount increased with the increase in concentration in the initial stage. However, with the further increase in concentration, the adsorption amount gradually stabilized, indicating the adsorption reached the equilibrium. The fitting curves and the parameters obtained from fitting two isotherm models with the experimental isotherm data are shown in Figure 4b,c and Table 2. The results indicated that the adsorption data better fit the Langmuir isotherm (R^2^ = 0.99), which means that the uptake of the acids was dependent on the monolayer adsorption model.

#### 2.2.5. Exploration of Desorption Conditions

**Figure 5:**
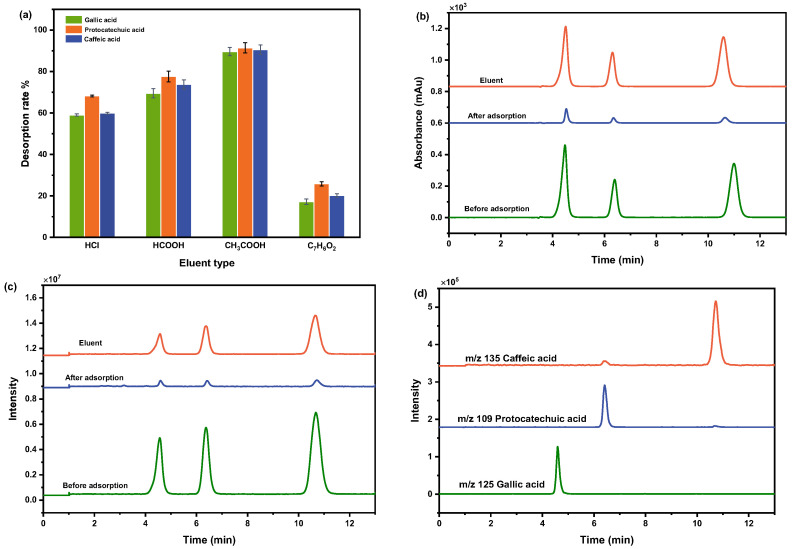
(**a**) Desorption rates of different desorption solutions; (**b**) HPLC chromatograms; (**c**) TIC images of before adsorption, after adsorption and desorption solution; (**d**) EIC images of desorption solution (*m*/*z* = 125, 109, 135).

Four pH 2 acidic solutions of hydrochloric acid, formic acid, acetic acid, and benzoic acid were selected to investigate the desorption effect and the desorption rate was chosen as the evaluation criterion. After 20 mg of adsorbent was added to the 8 mL mixture solution (0.5 mg/mL) for 2 h, the solid and liquid were separated and the solid was washed 3 times by adding 10 mL of water. Then, 8 mL each of different desorption solutions was added and shaken for 2 h. The HPLC method was used to detect the concentrations of various phenolic acids in the supernatant, and the desorption rate was calculated. As the results show in Figure 5a, the desorption rate was in the following order: CH_3_COOH, HCOOH, HCl, and benzoic acid. Among them, CH_3_COOH desorbed more than 90% of the phenolic acids and was used as the desorption solution.

The three phenolic acids were also analyzed using HPLC-MS before adsorption, after adsorption, and after desorption. The HPLC chromatograms in Figure 5b show that the peak areas of all the substances decreased after adsorption, indicating the presence of good adsorption. The total ion chromatography (TIC) of the MS in Figure 5c shows consistent peak times for the three substances before adsorption, after adsorption and desorption, indicating that all three substances retained their original structures. The extracted ion chromatograms (EIC) with *m*/*z* 125, *m*/*z* 109 and *m*/*z* 135 in Figure 5d were extracted, respectively, corresponding to the retention times on HPLC chromatograms, which indicated that all three substances were effectively desorbed. The TIC and EIC images obtained by MS analysis proved that the desorption components still kept their structures.

### 2.3. Comparison of SiO_2_-NH_2_-FPBA with the Reported Adsorbents

The adsorption capacities of SiO_2_-NH_2_-FPBA to phenolic acids were compared with amino functionalized silica gel (SiO_2_-NH_2_). The results are shown in Table 3. Under the same adsorption conditions, the adsorption capacities of SiO_2_-NH_2_ to CA, PCA, GA were apparently lower than those of SiO_2_-NH_2_-FPBA. This indicated that both the amino group and phenylboronic acid group contributed to the absorption of phenolic acids. They were also compared with other reported adsorbents (see Table 3). The results showed that SiO_2_-NH_2_-FPBA had faster adsorption kinetics and higher adsorption capacity than these reported adsorbents, which indicated its high potential for capturing phenolic acids.

### 2.4. Evaluation of the Adsorption of SiO_2_-NH_2_-FPBA to Phenolic Acids in C. spicatus Crude Extract

#### 2.4.1. Effect of pH on Absorption

**Figure 6:**
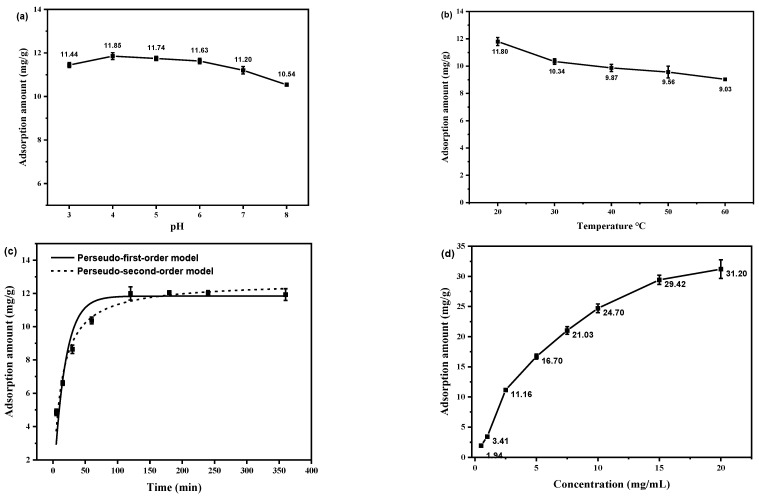
Effect of different factors on the adsorption capacity of phenolic acids from *C. spicatus* crude extract. (**a**) pH; (**b**) Temperature; (**c**) Time; (**d**) Concentration.

To explore the effect of pH, *C. spicatus* solutions were adjusted to 3, 4, 5, 6, 7, and 8 using HCl or NaOH, respectively. SiO_2_-NH_2_-FPBA absorbents of 50 mg were added into 4 mL of 2.5 mg/mL of each solution at different pH values. Batch adsorption experiments were conducted in a temperature-controlled water bath oscillator at 200 rpm at 20 °C for 4 h. At the end of the adsorption, the supernatant was filtered using a 0.45 μm filter membrane and the total phenol content was determined according to the Folin–Ciocalteu method. The adsorption effect at different pH values is shown in Figure 6a. It could be observed that the adsorption effect showed an increasing trend with increasing pH. The adsorption increased when the pH increased from 3 to 4, and reached a maximum value of 11.85 mg/g at pH 4. Then, as the pH increased from 4 to 8, the adsorption showed a decreasing trend. The pH of the aqueous solution of *C. spicatus* crude extract was 6.28. The adsorption amount at pH 6 was about 11.63 mg/g, which was only slightly different from the maximum value. Due to the complex phenolic acid composition in the crude extracts of *C. spicatus*, in order to better ensure its structure, all of the following adsorptions were performed using aqueous solutions of *C. spicatus* crude extracts.

#### 2.4.2. Effect of Temperature on Absorption

To explore the effect of adsorption temperature, 50 mg of SiO_2_-NH_2_-FPBA was added into 4 mL of crude extracts of aqueous *C. spicatus* (2.5 mg/mL). The centrifuge tubes were transferred into a water bath at different temperatures (20, 30, 40, 50, or 60 °C). After adsorption, the supernatant samples were filtered and measured, and the results are shown in Figure 6b. The results showed a significant decrease in adsorption with increasing temperature, which was presumed to be due to the fact that high temperature decomposed some of the substances in the crude extracts solution, thus reducing the adsorption effect. Finally, 20 °C was chosen for adsorption.

#### 2.4.3. Effect of Time on Absorption

To explore the adsorption kinetics, 50 mg of SiO_2_-NH_2_-FPBA was added into 8 mL of 2.5 mg/mL of each solution at 20 °C. The supernatant was sampled at 5, 15, 30, 60, 120, 180, 240, and 360 min. The effect of time on adsorption was investigated by detecting the concentration in the supernatant at different adsorption times, and the results are shown in Figure 6c. It could be seen that the adsorption amount tended to increase with time from 0 to 120 min, but as the time increased up to 360 min, the adsorption amount stabilized and reached 11.92 mg/g, indicating that the adsorption reached the maximum amount. Therefore, the optimal adsorption time was set to 2 h.

Pseudo-first-order and pseudo-second-order kinetic models were utilized to fit the adsorption process and fitting curves are shown in Figure S1a,b. It was found that the pseudo-second-order model had a better agreement (R^2^ = 0.99), which showed the process was a chemical adsorption.

#### 2.4.4. Effect of Concentration on Absorption

To explore the adsorption equilibrium, the solution was prepared as 0.5, 1, 2.5, 5, 7.5, 10, 15, and 20 mg/mL. Adsorbents of 50 mg were added into 4 mL of different concentrations of solution at 20 °C for 2 h, and the results are shown in Figure 6d. We determined that the adsorption amount tended to increase gradually with the increase in initial concentration. However, when the initial concentration increased from 15 mg/mL to 20 mg/mL, the adsorption amount stabilized, indicating that the adsorption reached saturation at this concentration.

The Langmuir and Freundlich models were adopted to describe the adsorption of SiO_2_-NH_2_-FPBA to phenolic acid substances. The fitting curves obtained from fitting two isotherm models with the experimental isotherm data are shown in Figure S2a,b. The results indicated the adsorption data better fit the Langmuir isotherm (R^2^ = 0.99) and the maximum theoretical adsorption capacity was 34.97 mg/g, which meant that the uptake of the phenolic acid substances was dependent on the monolayer adsorption model.

#### 2.4.5. Desorption Conditions

After adsorption of 50 mg of SiO_2_-NH_2_-FPBA to 4 mL of mixture solution (2.5 mg/mL) for 2 h, the solid and liquid were separated and the solid was washed 3 times by adding 10 mL of water. Then, 10 mL each of different desorption solutions—HCl, HCOOH, CH_3_COOH, and benzoic acid—was added and shaken for 2 h to calculate the desorption rate. The desorption rates were 72.60%, 56.43%, 42.21%, and 9.31% in the order of CH_3_COOH, HCOOH, HCl and benzoic acid, which showed the same trend as the previous desorption of phenolic acid simulants. However, the desorption rate was not as high as that of the previous. It was speculated that due to the complexity of the phenolic acid species in the crude extracts of *C. spicatus*, it was not guaranteed that each phenolic acid substance could be desorbed under the current conditions. A high desorption rate (>90%) needed more desorption solution (>20 mL).

**Figure 7:**
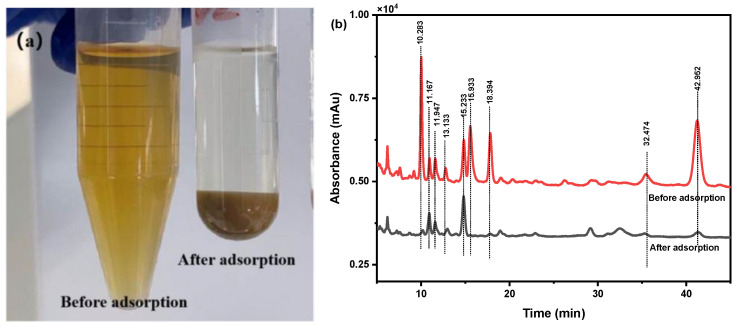
(**a**) Image of *C. spicatus* solution before and after adsorption; (**b**) HPLC of *C. spicatus* solution before and after adsorption.

A solution of 10 mg/mL of *C. spicatus* crude extracts was prepared. Then, 200 mg of SiO_2_-NH_2_-FPBA was added to 2.5 mL of *C. spicatus* solution (10 mg/mL) and the adsorption was shaken at room temperature for 2 h. The solutions before and after adsorption were determined using the Folin–Ciocalteu and HPLC methods. After adsorption, the removal rate was 93.24%, indicating that the vast majority of phenolic acids were adsorbed by SiO_2_-NH_2_-FPBA. As can be seen from Figure 7a, the color of *C. spicatus* before adsorption was brown and the supernatant after adsorption was almost transparent, indicating that SiO_2_-NH_2_-FPBA had a good adsorption effect on phenolic acids in the crude extracts of *C. spicatus*.

The solutions before and after adsorption were analyzed by using the HPLC method, and the results are shown in Figure 7b. It could be seen that several peak areas were significantly reduced, which were adsorbed by SiO_2_-NH_2_-FPBA. The peaks at 10.283 min, 15.933 min, and 18.394 min disappeared, and the peaks at 11.947 min, 13.133 min, 32.474 min, and 42.952 min were significantly reduced, indicating that the phenolic acid substances in the crude extracts of *C. spicatus* were successfully isolated by SiO_2_-NH_2_-FPBA.

### 2.5. Identification of the Separated Phenolic Acids by Q Exactive HF LC-MS

The compounds separated by SiO_2_-NH_2_-FPBA were analyzed by Q Exactive HF LC-MS. The results showed that twenty-two kinds of phenolic acids were successfully isolated (see Table 4). SiO_2_-NH_2_-FPBA could avoid most of the other hydroxy acids and could conveniently and quickly capture phenolic acids from the crude extract of *C. spicatus*. Thus, it is a good absorbent as the critical first step in the separation of phenolic acids.

### 2.6. Antioxidant and Hypoglycemic Activities of the Isolated Phenolic Acids from C. spicatus

**Figure 8:**
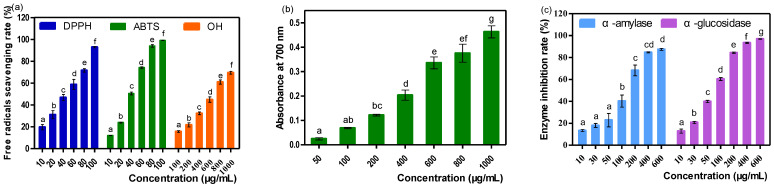
(**a**) The scavenging ability of the isolated phenolic acids from *C. spicatus* on DPPH, ABTS and hydroxyl radicals; (**b**) FRAP of the isolated phenolic acids from *C. spicatus*; (**c**) The α-amylase and α-glucosidase inhibition activity of the isolated phenolic acids from *C. spicatus*. Note: Different lowercase letters indicate significant differences (*p* < 0.05) among the columns with same color.

The antioxidant and hypoglycemic activities of the isolated phenolic acids from *C. spicatus* were studied by in vitro models. The results of the DPPH, ABTS and hydroxyl radicals scavenging assays and ferric-reducing antioxidant power (FRAP) assay are shown in Figure 8a,b.

The half maximal inhibitory concentration (IC_50_) values of the phenolic acids on DPPH, ABTS, and hydroxyl radicals were 36.07 ± 1.23 μg/mL, 32.63 ± 0.41 μg/mL and 600.21 ± 10.84 μg/mL, respectively, while the IC_50_ values of the positive group (ascorbic acid) were 4.63 ± 0.26 μg/mL, 6.02 ± 0.04 μg/mL and 298.13 ± 7.32 μg/mL, respectively. Furthermore, the phenolic acids presented a dose-dependent FRAP as well as ascorbic acid. In addition, the FRAP of phenolic acids (1 mg/mL) was approximately identical to that of ascorbic acid (123.33 μg/mL).

The results of the α-amylase and α-glucosidase inhibitory assays are shown in Figure 8c. The results showed that phenolics acids from *C. spicatus* presented a great inhibitory effect against α-amylase and α-glucosidase, with IC_50_ values of 110.63 ± 3.67 μg/mL and 64.76 ± 0.30 μg/mL, respectively. As for the positive group (acarbose), the IC_50_ values were 0.342 ± 0.019 mg/mL and 8.986 ± 0.464 ng/mL, respectively.

## 3. Materials and Methods

### 3.1. Materials

Silica gel for thin-layer chromatography, used as the support material of the adsorbent, was purchased from a branch of Qingdao Haiyang Chemical Plant (Qingdao, China). 3-Aminopropyl triethoxysilane (3-APTES, 98%), 4-formylbenzoic acid (4-FPBA, 97%), caffeic acid (CA, 98%), protocatechuic acid (PCA, 97%), disodium hydrogen phosphate (Na_2_HPO_4_, 99%), and sodium dihydrogen phosphate (NaH_2_PO_4_, 97%) were obtained from Heowns Biochemical Technology (Tianjin, China). Gallic acid (GA, 99%) was from Macklin Biochemical (Shanghai, China). Sodium cyanoborohydride (NaBH_3_CN, 95%) was from Meryer Chemical Technology Co., Ltd. (Shanghai, China). Anhydrous ethanol and sodium carbonate (Na_2_CO_3_, 99.8%) were obtained from Jiangtian Chemical Technology (Tianjin, China). Methanol was from Aladdin Chemical Technology (Shanghai, China). Formic acid (HCOOH, 88%) was from Kermel Chemical Technology (Tianjin, China). Acetic acid (CH_3_COOH, 99.5%) was from Jieerzheng Chemical (Tianjin, China). Hydrochloric acid (HCl, 36–38%) was from Rionlon Pharmaceutical Chemical (Tianjin, China). Benzoic acid (C_7_H_6_O_2_, 99.5%) and sodium hydroxide (NaOH, 96%) were obtained from Damao Chemical Reagents (Tianjin, China). Catechol (99.5%) was from Rhawn Chemical Technology (Shanghai, China). Folin–Ciocalteu reagent (1 mol/L) was from Yuanye Biochemical (Shanghai, China). *Clerodendranthus spicatus* (*C. spicatus*) was obtained from the Xishuangbanna region (Yunnan, China.) 1,1-Diphenyl-2-picrylhydrazyl (DPPH) and 2,2′-azinobis-(3-ethylbenthiazoline-6-sulfonate) (ABTS) were from Sigma-Aldrich (St. Louis, MO, USA). α-Amylase (3700 U/g) was from Solarbio Science & Technology Co., Ltd. (Beijing, China). α-Glucosidase (50 U/mg) was from Yuanye Biochemical (Shanghai, China). Other reagents and chemicals were of analytical grade and obtained locally.

### 3.2. Preparation of Phenylboronic Acid and Amino Bifunctional Modified Absorbent

#### 3.2.1. Preparation of Amino Modified Silica Gel (SiO_2_-NH_2_)

Fifty grams of silica gel was dispersed in 200 mL of HCl (6 mol/L) and stirred at 60 °C. After 6 h, it was taken out and washed with ultrapure water to neutral pH and dried in a vacuum oven overnight. The silica gel was activated in a vacuum oven at 130 °C for 3 h. Ten grams of activated silica gel was dispersed in 50 mL anhydrous toluene. After adding 12 mL of 3-APTES, the mixture was stirred for 24 h at 110 °C. The product was washed by toluene, anhydrous ethanol, and acetone, in turn, three times. After drying, SiO_2_-NH_2_ was obtained.

#### 3.2.2. Preparation of Phenylboronic Acid and Amino Modified Adsorbent (SiO_2_-NH_2_-FPBA)

**Figure 9:**
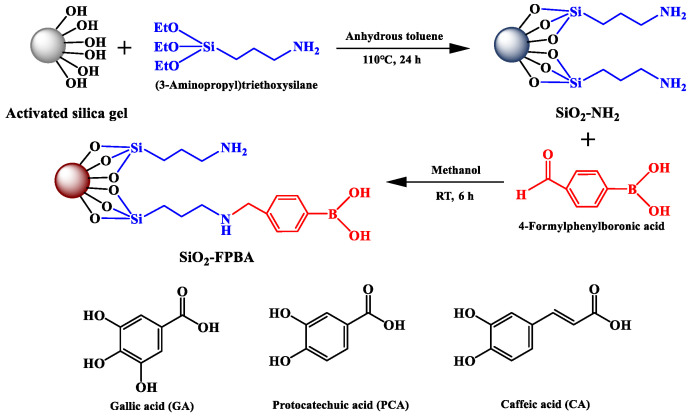
Synthesis route of SiO_2_-NH_2_-FPBA and structures of GA, PCA and CA.

One gram of SiO_2_-NH_2_ was weighed and transferred into 50 mL methanol. After adding 0.48 g of 4-FPBA (3.18 mmol) and 0.4 g of NaCNBH_3_ (6.36 mmol), the mixture was stirred at 500 rpm for 6 h at 20 °C. After the reaction, the solid product was filtered and washed with ethanol and water, and dried under vacuum at 60 °C for 6 h to obtain the SiO_2_-NH_2_-FPBA. The optimization of the reaction conditions, including the amount of 4-FPBA input, the amount of reducing agent, the reaction time and temperature, is shown in Table S1. The synthesis route is shown in Figure 9.

### 3.3. Adsorption Experiments on GA, CA and PCA

GA, CA and PCA were selected as the target compounds in the adsorption experiments (structures are shown in Figure 9). In order to optimize the phenolic acids’ adsorption conditions, the effects of initial pH, temperature, time and concentration were investigated. The GA, CA, and PCA solution was, respectively, mixed with 20 mg of adsorbents at different pH values (3–8) with different concentrations (0.1–5 mg/mL) and temperature (20–60 °C) for different durations (5–420 min). At the end of the adsorption, the solid was separated from the liquid by centrifugation at 4100 rpm for 3 min. The concentration of the GA, CA, and PCA solution in the supernatant was measured by UV–vis spectrometry at 264 nm, 290 nm and 320 nm, respectively.

The amount of each phenolic acid adsorbed (mg/g) was calculated by the following Equation (5):

(5) $$Q=\frac{\left(C_{0}-C_{e}\right)\times V}{m}$$

where C_0_ and C_e_ are the initial and equilibrium phenolic acid solutions (mg/mL), respectively, V is the volume (mL) of initial solution and m is the mass (g) of adsorbent.

To simulate the environment of the phenolic acids mixture, the solution was prepared by mixing GA, CA, and PCA. The mixed solution contained 0.5 mg/mL of GA, 0.5 mg/mL of CA and 0.5 mg/mL of PCA, respectively. The concentration of each substance in the mixture before adsorption, after adsorption and the desorption solution was determined by HPLC, which was performed on a Chuangxintongheng LC3000 system (Beijing, China); HPLC-MS was performed on a 1260 Infinity UHPLC-6420 system (Agilent, Santa Clara, CA, USA). HPLC conditions were generally as follows: The column was a Baulo C18 (250 × 4.6 mm, 5 μm) from Tianjin Aumi Science & Technology Co., Ltd. (Tianjin, China) The column temperature was room temperature. The mobile phase was methanol and 0.1% formic acid (*v*/*v*, 30:70). The flow rate was 1 mL/min. The sample injection volume was 5 μL. The detection wavelength was at 280 nm. The HPLC conditions in HPLC-MS were the same as above. Mass spectrum conditions were as follows: Effluents were determined by ESI combined with multiple reaction monitoring (MRM) in negative ion mode. The fragmentation voltage was 135 V, the nebulizer gas was nitrogen, and the gas flow rate was 7 mL/min. The quantitative ion pairs of the GA, CA, and PCA to be measured were *m*/*z* 169 → 125, *m*/*z* 179 → 135, and *m*/*z* 153 → 109. After detecting the concentrations of various phenolic acids in the supernatant, the desorption rate was calculated according to the following Equation (6):

(6) $$Desorption\;rate=\frac{C_{d}\times V_{d}}{V\times \left(C_{0}-C_{e}\right)}\times 100\%$$

where C_d_ and V_d_ are the concentration (mg/mL) and the desorption volume of each phenolic acid (mL), respectively. V, C_0_ and C_e_ are the same as Equation (5).

### 3.4. Adsorption of SiO_2_-NH_2_-FPBA to Phenolic Acids from C. spicatus Extract Solutions

The dried *C. spicatus* was ground with a grinding machine and screened through a 30-mesh sifter. The powder was mixed with 50% (*v*/*v*) ethanol at the solid–liquid ratio of 1:25. Ultrasonic extraction was carried out for 45 min twice. After filtration, the supernatant was concentrated with a rotary evaporator to obtain the crude extracts of *C. spicatus.* The effects of initial pH, temperature, time and concentration were investigated to optimize the adsorption of SiO_2_-NH_2_-FPBA to phenolic acids from crude extract solutions of *C. spicatus*. Then, 50 mg of SiO_2_-NH_2_-FPBA was added into *C. spicatus* crude extract solutions at different pH values (3–8) with different concentrations (0.5–20 mg/mL) and temperatures (20–60 °C) for different durations (5–360 min). The adsorption amount was calculated according to Equation (5), where C was the concentration of the total phenolic acids content. The Folin–Ciocalteu method was used to determine the total phenolic acids content.

The Folin–Ciocalteu reagent can oxidize the phenolic acids to show a blue color, and the shade of blue is proportional to the phenolic acids content. GA was selected as a standard substance. GA solutions of 1 mg/mL were diluted to obtain 0.01, 0.015, 0.02, 0.025, and 0.03 mg/mL of GA solutions. Then, 1 mL of the above solutions were added to 0.5 mL of Folin–Ciocalteu reagent, 1 mL of 12% Na_2_CO_3_ solution and 2.5 mL of water. After mixing well and being left to stand for 45 min in a 45 °C water bath, the absorbance of the solution at 765 nm was measured to establish the standard curve. The *C. spicatus* crude extract solution to be tested after adsorption was processed by the same method and measured at 765 nm.

Solutions of crude extract of *C. spicatus* before and after adsorption were analyzed by HPLC. The column was a Baulo C18 (250 × 4.6 mm, 5 μm) and the column temperature was at room temperature. The mobile phase was methanol and 0.1% formic acid solution (*v*/*v*, 15:85). The flow rate of the mobile phase was 1 mL/min and the sample injection volume was 5 μL. The column effluent was monitored at a wavelength of 280 nm.

### 3.5. Identification of the Separated Phenolic Acids by Q Exactive HF LC-MS

The isolated substances were determined by Q Exactive HF LC-MS (Thermo Fisher Scientific, Waltham, MA, USA). The conditions were as follows: The column was an Agilent C18 (4.6 ID × 250 mm). The column temperature was at room temperature. The mobile phase was methanol and 0.1% formic acid. The gradient procedure was 40–95% methanol at 0–60 min and 95% methanol at 60–80 min at a flow rate of 0.4 mL/min. The injection volume was 20 μL. The detection wavelength was 280 nm. ESI combined with the anion mode was used for the determination of MS.

### 3.6. The Antioxidant and Hypoglycemic Activities of the Isolated Phenolic Acids In Vitro

The antioxidant capacity of the isolated phenolic acids from *C. spicatus* was determined by DPPH, ABTS and hydroxyl radicals scavenging assays and FRAP.

DPPH, ABTS and hydroxyl radicals scavenging activities were determined according to our previous methods with ascorbic acid as the positive control [42]. The results were assessed as follows:

Inhibition (%) = [(A_blank_ − A_sample_)/A_blank_] × 100% (7)

where A_sample_ and A_blank_ are the UV absorbance of the sample and blank, respectively.

FRAP was evaluated according to our previous study [43]. The hypoglycemic capacities of the phenolic acids from *C. spicatus* were measured by α-amylase and α-glucosidase inhibitory assays with acarbose as the positive control [44]. The calculations of inhibition on α-amylase and α-glucosidase were the same as Equation (7).

### 3.7. Statistical Analysis

Statistical analysis was used to analyze the results. Data were expressed as mean ± standard deviation (SD) and IC_50_ calculations were performed using SPSS version 22.0. A one-way ANOVA test (Tukey’s test) was used to determine significant statistical differences. The means were regarded as significantly different at *p* < 0.05.

## 4. Conclusions

A phenylboronic acid and amino bifunctional modified silica gel SiO_2_-NH_2_-FPBA was synthesized and applied to the separation of GA, CA, PCA and phenolic acids in *C. spicatus* crude extracts. FT-IR, BET, SEM, EDS, TEM, TGA and ICP-OES characterizations were conducted to verify its structure. The adsorption experiments showed that the adsorption amounts of 5 mg/mL of gallic acid, caffeic acid and protocatechuic acid aqueous solution at 20 °C were 103.22 mg/g, 115.49 mg/g, and 109.97 mg/g. The three substances all followed the pseudo-second-order kinetic model, and reached adsorption equilibrium after 120 min. The adsorption isotherm was fit the Langmuir model, which indicated a monolayer adsorption. CH_3_COOH at pH 2 could desorb 90% of the phenolic acids. HPLC-MS results showed that the eluted substances still maintained their original structures. The adsorption amount of SiO_2_-NH_2_-FPBA to phenolic acids in crude extracts of *C. spicatus* could reach 31.20 mg/g for 2 h at 20 °C. The adsorption kinetics followed the pseudo-second-order model and the adsorption isotherm followed the Langmuir model. The HPLC analysis of the crude extract solutions of *C. spicatus* before and after the adsorption showed that the peak areas at different retention times were observably reduced and the adsorption rate was 93.24%. Twenty-two kinds of phenolic acids were identified by Q Exactive HF LC-MS, which indicated that SiO_2_-NH_2_-FPBA had a significant effect on the one-step isolation of phenolic acid substances from *C. spicatus*. Furthermore, the isolated phenolic acids from *C. spicatus* possessed great antioxidant activity with DPPH, ABTS, and hydroxyl radicals scavenging activity (IC_50_ = 36.07 ± 1.23 μg/mL, 32.63 ± 0.41 μg/mL and 600.21 ± 10.84 μg/mL, respectively) and ferric-reducing power. Furthermore, the phenolic acids showed effective inhibition on α-amylase and α-glucosidase (IC_50_ = 110.63 ± 3.67 μg/mL and 64.76 ± 0.30 μg/mL, respectively). The findings demonstrate that SiO_2_-NH_2_-FPBA has significant potential in the practical application of separating active ingredients’ constituents from natural resources for traditional Chinese medicine.

## Figures and Tables

**Table 1:** Pseudo-first-order and pseudo-second-order adsorption kinetics model parameters.

Phenolic Acid Type	Pseudo-First-Order			Pseudo-Second-Order		
	k_1_ (min^−1^)	Q_m_ (mg/g)	R^2^	k_1_ (g·mg^−1^min^−1^)	Q_m_ (mg/g)	R^2^
Gallic acid	0.0007246	37.13	0.5330	0.006863	76.45	0.9999
Caffeic acid	0.002280	27.81	0.6038	0.002738	93.28	0.9999
Protocatechuic acid	0.001680	35.55	0.5832	0.002461	85.94	0.9992

**Table 2:** Langmuir and Freundlich isotherm models and parameters.

Phenolic Acid Type	Langmuir Parameters			Freundlich Parameters		
	Q_0_	K_L_	R_L_^2^	K_F_	n	R_F_^2^
Gallic acid	105.7	8.298	0.9989	83.19	4.736	0.9573
Caffeic acid	116.2	13.48	0.9989	100.2	9.109	0.9889
Protocatechuic acid	111.6	8.960	0.9987	90.25	6.517	0.9943

**Table 3:** Comparison of phenolic acid adsorption properties with other reported adsorbents.

Adsorbent	Compounds *	Adsorption Capacity (mg/g)	Time (min)	Reference
SILs	FA, CA, SA	53.2–72.2	-	[12]
Fe_3_O_4_@SiO_2_	CA	21.10	35	[18]
MCMD	GA, CA	31.95, 27.64	720	[19]
AMCS	GA	48.38	720	[20]
Fe_3_O_4_@NH_2_ (CB [6]) NH_2_ MNPs	SAB, FA, RA, SAA, LA, CA	21.96–36.36	25	[39]
pH-MMIPs	CA	11.50	-	[40]
NKA-II resin	CGA	66.86	300	[41]
SiO_2_-NH_2_	CA, PCA, GA	81.19, 38.15, 58.45	120	This work
SiO_2_-NH_2_-FPBA	CA, PCA, GA	93.64, 89.35, 79.66	120	This work

* FA: ferulic acid; CA: caffeic acid; SA: salicylic acid; GA: gallic acid; SAB: salvianolic acid B; RA: rosmarinic acid; SAA: salvianolic acid A; LA: lithospermic acid; CGA: chlorogenic acid; PCA: protocatechuic acid.

**Table 4:** Identification of phenolic acid compounds adsorbed by SiO_2_-NH_2_-FPBA.

No.	Rt (min)	Compound Identified	Molecular Formula	Molecular Weight	[M-H]^−^ *m*/*z*	Err [ppm]	MS/MS
1	6.73	Dihydroferulic acid	C_10_H_12_O_4_	196.0736	195.0658	3.007	195.05064, 59.01374
2	6.85	Chlorogenic acid	C_16_H_18_O_9_	354.0945	353.1023	−0.095	191.03650
3	6.93	Salicylic acid	C_7_H_6_O_3_	138.0313	137.0241	1.119	119.02329, 93.03446
4	7.99	2,5-Dihydroxybenzaldehyde	C_7_H_6_O_3_	138.0317	137.0241	4.017	137.02419, 109.02918
5	8.10	Gallic acid	C_7_H_6_O_5_	170.0212	169.1230	1.325	125.02416
6	8.14	Rosmarinic acid	C_18_H_16_O_8_	360.0845	359.2044	1.475	197.04518, 179.03462, 161.02415, 135.04495, 123.04496, 72.99299
7	8.17	Succinic acid	C_4_H_6_O_4_	118.0266	117.3994	4.574	117.01916, 114.40305, 99.00867, 73.02940
8	8.24	4-Hydroxybenzaldehyde	C_7_H_6_O_2_	122.0368	121.0292	4.663	121.02927, 108.02168, 93.03434
9	8.88	3,5-*O*-dimethyl gallic acid	C_9_H_10_O_5_	198.0528	197.8075	2.651	197.80759
10	10.42	Caffeic acid ethyl ester	C_11_H_12_O_4_	208.0736	207.1385	2.834	207.02722, 179.03477, 135.04494
11	10.62	Protocatechuic acid	C_7_H_6_O_4_	154.0267	153.0190	4.154	153.01906, 109.02934
12	10.64	Dihydrocaffeic acid	C_9_H_10_O_4_	182.0579	181.0502	2.964	181.05038, 137.06052, 109.02934, 59.01374
13	11.26	Caffeic acid	C_9_H_8_O_4_	180.0423	179.1928	3.276	135.04494
14	11.29	Vanillic acid	C_8_H_8_O_4_	168.0423	167.0459	3.510	167.033474, 152.01125, 138.92929, 108.02152
15	12.07	Protocatechuic aldehyde	C_7_H_6_O_3_	138.0317	137.5061	4.017	137.02415
16	18.76	Yunnaneic acid D	C2_7_H_24_O_12_	540.1267	539.1385	0.875	491.10147, 297.07635, 179.03484, 161.02408, 135.04500
17	20.31	Rosmarinic acid-Glc	C_24_H_26_O_13_	522.1373	521.1290	0.973	359.09784, 341.08820, 323.07614, 197.04514, 179.03461
18	21.38	Salvianolic aid A	C_26_H_22_O_10_	494.1140	493.2072	0.763	295.06058, 267.06546, 203.03455, 185.02414, 135.04495, 109.02928
19	23.22	Dicaffeoyltartaric acid	C_22_H_18_O_12_	474.0798	473.0689	1.102	179.03491, 161.02411, 135.04504
20	23.99	Lithospermic acid	C_27_H_22_O_12_	538.1111	537.1029	0.971	493.11276, 161.02406, 135.04492
21	24.35	Clerodendranoic acid	C_29_H_26_O_12_	566.1424	565.1553	0.923	357.09732
22	64.45	Asiatic acid	C_30_H_48_O_5_	488.3493	487.3419	−0.668	487.34143

## Data Availability

All data are available from the authors.

## Supplementary Materials

The following supporting information can be downloaded at: https://www.mdpi.com/article/10.3390/molecules28227539/s1, Figure S1: (a) Fitted curves of pseudo-first-order model; (b) pseudo-second-order model of the adsorption of *C. spicatus*; Figure S2: (a) Fitted curves of Langmuir model; (b) Freundlich model of the adsorption of *C. spicatus*. Table S1: Preparation and conditions optimization of SiO_2_-NH_2_-FPBA;
