# Supplementary material for: Phenylboronic Acid and Amino Bifunctional Modified Adsorbent for Quickly Separating Phenolic Acids from Crude Extract of Clerodendranthus spicatus and Evaluation of Their Antioxidant and Hypoglycemic Activities
Source: Molecules. 2023 Nov 11;28(22):7539. doi: 10.3390/molecules28227539 (PMC10673389; doi:10.3390/molecules28227539)
Supplement: Supplementary file 1 [file molecules-28-07539-s001.zip › molecules-2682867-supplementary.pdf]

Supplementary Materials

Phenylboronic Acid and Amino Bifunctional Modified Adsorbent for Quickly Separating Phenolic Acids from Crude Extract of *Clerodendranthus spicatus* and Evaluation of Their Antioxidant and Hypoglycemic Activities

Mengqi Cheng, Qianyi Song, Xiaoyu Zhang, Pingyi Zheng, Ran Zhao, Youxin Li*, and Haixia Chen*

Preparation and conditions optimization of SiO_2-NH_2-FPBA

The conditions including the amount of 4-FPBA input, the amount of reducing agent, the reaction time and temperature for the synthesis of SiO_2-NH_2-FPBA were optimized. The phenylboronic acid content was used as a criterion for judging whether the reaction was optimized or not. The determination principle of the boronic acid group is that boronic acid can bind specifically with cis-diols group. Catechol has a cis-diol structure and has a maximum absorption wavelength at 275 nm, which can be used for quantitative calculations. In detail, 0.35 mg/mL catechol solution was prepared with 1/15 mol/L pH 8.5 NaH_2PO_4-Na_2HPO_4 solution. All the processes were protected from light. SiO_2-NH_2-FPBA of 25 mg and 10 mL catechol solution of 0.35 mg/mL were added into centrifuge tube and shaken for 4 h. After adsorption, the supernatant solution was diluted and determined by UV-Vis spectrophotometer. A series of different concentration catechol standard solutions (0.014, 0.0175, 0.021, 0.0245, 0.028, and 0.035 mg/mL) were prepared for the standard curve. All the processes were protected from light and all experiments were in parallel three times.

The content of the phenylboronic acid group (mmol/g) was calculated by the following Eq. (1):

$$Q = \frac{(C_0 - C_1)}{m \times M} \quad (1)$$

where C_0 and C_e are the initial and equilibrium catechol solution concentrations (mg/mL), respectively. V is the volume (mL) of catechol solution and m is the mass (g) of SiO_2-NH_2-FPBA. M is the molar mass (g/mol) of catechol.

In order to make the SiO_2-NH_2 (amino content is 2.1170 mmol/g) fully react with the aldehyde group, 4-FPBA was put in molar ratios of 1:1, 1:1.5, and 1:2, and the input amounts were 2.12, 3.18, 4.24 mmol. The results were shown in the Table S1, entry 1-3. Phenylboronic acid content increased with the increase of reactants and the maximum amount of phenylboronic acid content was 0.4355 mmol/g at the ratio of 1:1.5. When the input amount of 4-FPBA was increased to 1:2, the phenylboronic acid content no longer increased significantly, and it was assumed that the amino group on the SiO_2-NH_2 had completely participated in the reaction. Therefore, a ratio of 1:1.5 was chosen for the subsequent experiments.

In the investigation of the reaction time, the reaction time was set to 6, 12, and 24 h. From the Table S1, entry 2, 4-5, it was observed that the phenylboronic acid content decreased slightly by increasing the reaction time. The highest phenylboronic acid content of 0.4391 mmol/g was obtained when the reaction time was 6 h. Therefore, 6 h was chosen as the reaction time to improve the efficiency of the reaction.

The amount of input of $NaBH_3CN$ was investigated (Table S1, entry 4, 6-7) according to the ratio of 4-FPBA:$NaBH_3CN$ as 1:1, 1:2, and 1:3. The results showed that when the input ratio was 1:2, the phenylboronic acid content was 0.4453 mmol/g. Too little reducing agent would lead to incomplete reduction and too much input would lead to waste, and 1:2 ratio was chosen.

The reaction temperatures were investigated and set at 20, 30, 40, and 50 ℃ (Table S1, entry 7-10). It was observed that the phenylboronic acid content decreased significantly with increasing temperature, from 0.4453 mmol/g to 0.3064 mmol/g, indicating that the reaction was not suitable for high temperature conditions. Thus, the reaction was chosen to be carried out at room temperature.

Table S1. Preparation and conditions optimization of SiO_2-NH_2-FPBA.

Entry	4-FPBA (mmol)	$NaBH_3CN$ (mmol)	Time (h)	Temperature (°C)	Phenylboronic acid content (mmol/g)
1	2.12	9.54	24	20	0.3242
2	3.18	9.54	24	20	0.4355
3	4.24	9.54	24	20	0.4213
4	3.18	9.54	6	20	0.4391
5	3.18	9.54	12	20	0.4330
6	3.18	3.18	6	20	0.4334
7	3.18	6.36	6	20	0.4453
8	3.18	6.36	6	30	0.4343
9	3.18	6.36	6	40	0.3660
10	3.18	6.36	6	50	0.3064

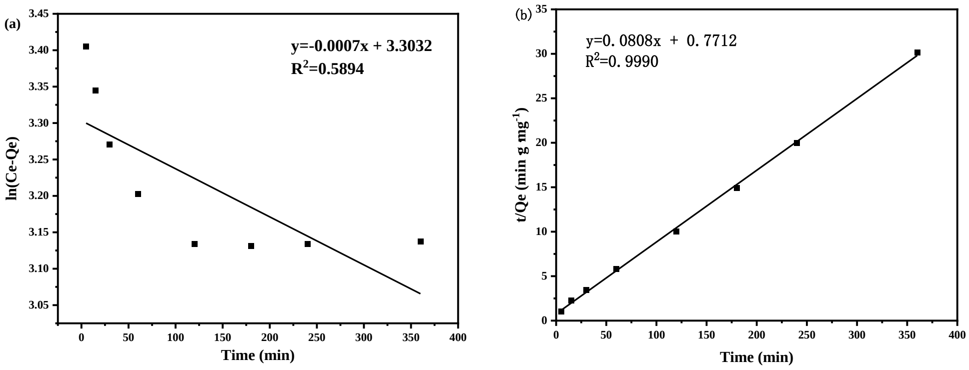

Figure S1. (**a**) Fitted curves of pseudo-first-order model; (**b**) pseudo-second-order model of the adsorption of *C. spicatus.*

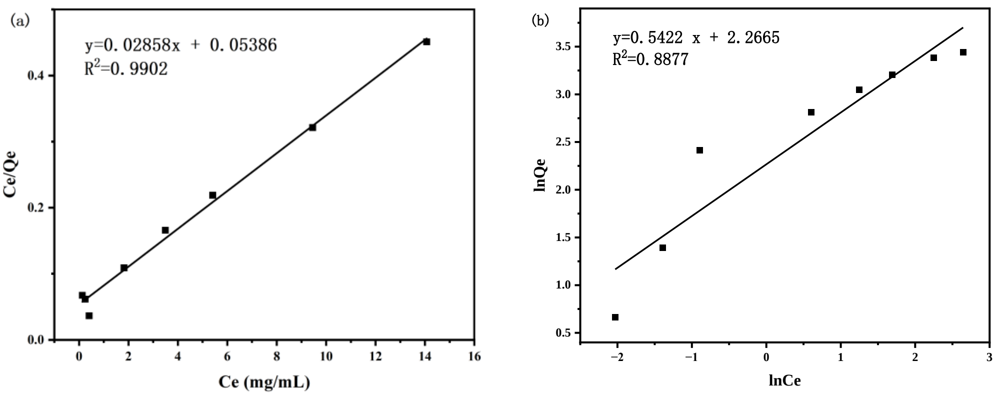

Figure S2. (**a**) Fitted curves of Langmuir model; (**b**) Freundlich model of the adsorption of *C.spicatus.*
